# Association of Single-Nucleotide Polymorphisms Rs2779249 (chr17:26128581 C>A) and Rs rs2297518 (chr17: chr17:27769571 G>A) of the *NOS2* Gene with Tension-Type Headache and Arterial Hypertension Overlap Syndrome in Eastern Siberia

**DOI:** 10.3390/genes14020513

**Published:** 2023-02-17

**Authors:** Polina V. Alyabyeva, Marina M. Petrova, Diana V. Dmitrenko, Natalia P. Garganeeva, Galina A. Chumakova, Mustafa Al-Zamil, Vera V. Trefilova, Regina F. Nasyrova, Natalia A. Shnayder

**Affiliations:** 1 Shared Core Facilities Molecular and Cell Technologies, V.F. Voino-Yasenetsky Krasnoyarsk State Medical University, 660022 Krasnoyarsk, Russia; 2 Department of General Medical Practice and Outpatient Therapy, Siberian State Medical University, 634050 Tomsk, Russia; 3 Department of Therapy and General Medical Practice with a Course of Postgraduate Professional Education, Altai State Medical University, 656038 Barnaul, Russia; 4 Department of Physiotherapy, Faculty of Continuing Medical Education, Peoples’ Friendship University of Russia, 117198 Moscow, Russia; 5 Neurological Department No. 16, Hospital for War Veterans, 193079 St. Petersburg, Russia; 6 Institute of Personalized Psychiatry and Neurology, V.M. Bekhterev National Medical Research Center for Psychiatry and Neurology, 192019 St. Petersburg, Russia

**Keywords:** hypertension, tension-type headache, overlap syndrome, iNOS, *NOS2*, biomarker, single-nucleotide polymorphism, rs2779249

## Abstract

Inducible nitric oxide (NO) synthase (iNOS), encoded by the *NOS2* gene, promotes the generation of high levels of NO to combat harmful environmental influences in a wide range of cells. iNOS can cause adverse effects, such as falling blood pressure, if overexpressed. Thus, according to some data, this enzyme is an important precursor of arterial hypertension (AH) and tension-type headache (TTH), which are the most common multifactorial diseases in adults. The purpose of this study was to investigate the association of rs2779249 (chr17:26128581 C>A) and rs2297518 (chr17: chr17:27769571 G>A) of the *NOS2* gene with TTH and AH overlap syndrome (OS) in Caucasians in Eastern Siberia. The sample size was 91 participants: the first group—30 patients with OS; the second group—30 patients AH; and the third group—31 healthy volunteers. RT-PCR was used for the determination of alleles and genotypes of the SNPs rs2779249 and rs2297518 of the *NOS2* gene in all groups of participants. We showed that the frequency of allele A was significantly higher among patients with AH compared with healthy volunteers (*p*-value < 0.05). The frequency of the heterozygous genotype CA of rs2779249 was higher in the first group vs. the control (*p*-value = 0.03), and in the second group vs. the control (*p*-value = 0.045). The frequency of the heterozygous genotype GA of rs2297518 was higher in the first group vs. the control (*p*-value = 0.035), and in the second group vs. the control (*p*-value = 0.001). The allele A of rs2779249 was associated with OS (OR = 3.17 [95% CI: 1.31–7.67], *p*-value = 0.009) and AH (OR = 2.94 [95% CI: 1.21–7.15], *p*-value = 0.015) risks compared with the control. The minor allele A of rs2297518 was associated with OS (OR = 4.0 [95% CI: 0.96–16.61], *p*-value = 0.035) and AH (OR = 8.17 [95% CI: 2.03–32.79], *p*-value = 0.001) risks compared with the control. Therefore, our pilot study demonstrated that the SNPs rs2779249 and rs229718 of the *NOS2* gene could be promising genetic biomarkers for this OS risk in Caucasians from Eastern Siberia.

## 1. Introduction

A single-nucleotide variant (SNV) is a change in the deoxyribonucleic acid (DNA) sequence that occurs when a single nucleotide is replaced in the genome sequence [1]. A single-nucleotide polymorphism (SNP) is a genetic variant that must be present in at least 1% of population [2]. The role of SNPs in the understanding of multifactorial disease (MFDs) pathogenesis, therapeutic response, and even ultimately personalized medicine will become indispensable in the future. SNPs determine differences in our predisposition to a wide range of diseases, so the search for genetic biomarkers is an extremely relevant scientific topic today [3]. The role of SNPs in the developmental, treatment and prognosis of common MFDs is being actively studied, including in cases of tension-type headache (TTH) [4,5] and arterial hypertension (AH) [4,6]. As known, MFDs are caused by complex interactions between endogenous (genetic) and exogenous (environmental) factors. The term “multifactorial” means that there are many different factors working together to cause the onset of these diseases. These include the effects of a combination of genetic factors, none of which by themselves can cause disease, and environmental factors, which, again, do not by themselves cause MFDs. The mutual influence of various combinations of genetic and environmental factors contributes to the development of MFDs [7].

TTH is a common MFD of the primary headache group in adults. In addition, AH is one of the main examples of cardiovascular MFDs in adults. Previous studies have shown that persons with TTH have a high risk of developing AH, and hypertensive patients have a high risk of developing TTH. These risks are higher than those of the general population [6]. 

Moreover, in the conditions of personalized medicine, it has become relevant to study not individual diseases, but their combination in case of comorbidity in one patient. In this case, we are talking about clinical phenotypes or overlap syndromes (OSs). As we know, OS is a medical condition which shares the features of at least two more widely recognized disorders [8], for example: TTH and AH [9,10]. Usually, OSs present with a subacute onset and they are heterogeneous as many combinations of clinical and genetic features can occur [8]. The relationship between TTH and AH is potentially of great pathophysiological and clinical interest as a genetic determination OS [9]. The studies of genetic predisposition to this OS are rare, and their results are contradictory [4], although the potential role of the alteration of nitric oxide (NO) synthesis and NO-synthase (NOSs) expression or/and functional activity in the development of OS is known [4,10]. 

**Figure 1:**
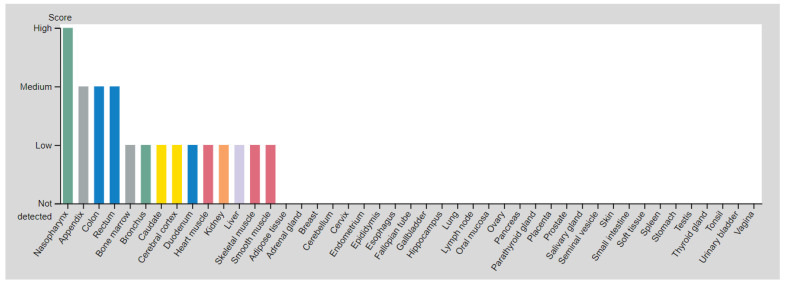
The inducible nitric oxide synthase (iNOS) expression in the healthy organs of the human body (https://www.proteinatlas.org/ENSG00000007171-NOS2/tissue accessed on 17 November 2022).

Inducible NO synthase (iNOS), encoded by the *NOS2* gene, promotes the generation of high levels of NO to combat harmful environmental influences in a wide range of cells [11]. iNOS expression in healthy heart and vessel tissues (Figure 1) is induced by inflammation or cardiac damage [12], by other various environmental factors and stress [13], in contrast to the constitutive enzymes’ neuronal and endothelial NOS. When overexpressed, iNOS can cause side effects, such as excessive lowering of blood pressure, as well as changes in skeletal muscle relaxation [14,15]. Thus, according to some data, this enzyme is an important precursor of OS [4,9], which are the most common MFDs in adults.

iNOS is encoded by the *NOS2* gene (17q11.2). It is probable that SNPs of the *NOS2* gene may be important biomarkers of OS (TTH and AH) in adults. Therefore, many SNPs have been described in this regard, and the most promising as biomarkers of this OS were four SNPs [4], including rs2779249 (chr17:26128581 C>A), rs2297518 (chr17: 26096597 G>A), rs1800482 (chr17:26128509 C>G), and rs3730017 (chr17:26109102 G>A) [16].

The role of rs2779249 and rs2297518 of the *NOS2* gene is being actively studied, however, these are only a few studies, for example, such associative genetic studies have been performed in population of Finland [17], Brazil [18], and China [19], but their results are contradictory. This testifies to the clinical-geographical, racial and ethnic heterogeneity of the samples and does not yet allow us to obtain a unanimous opinion about these SNPs as OS biomarkers. As far as we know, in Russia, which is characterized by a large extent of territory, and racial and ethnic heterogeneity of the population, no associative genetic studies of the role of the *NOS2* gene have been previously carried out.

Our scientific and clinical interest in these SNPs is also explained by the fact that the association of minor alleles with highest risk of stress [20] and depression [21], early vascular aging, quality of aging and longevity in humans [22,23], oxidative stress [24], neurodegeneration [25,26], and ischemic stroke [27] has previously been shown. Thus, these associations may partially explain the risk of adverse outcomes of this OS [10].

Thus, the results of previously conducted associative genetic studies indicate that genetic variants within the NO-forming pathway can change susceptibility to OS and worsen its clinical outcomes. At the same time, replication of these results in other independent cohorts may be justified.

The purpose of this study is to study the association of SNPs rs2779249 (chr17:26128581 C>A) and rs2297518 (chr17: chr17:27769571 G>A) of the *NOS2* gene with OS (TTH and AH) in Caucasians in Eastern Siberia.

## 2. Materials and Method

### 2.1. Data Collection

The conduct of our research was consistent with the principles reflected in the Helsinki Declaration [28]. All stages of this study were discussed and approved by the Ethics Committee of V.F. Voino-Yasenetsky Krasnoyarsk State Medical University (KrasSMU). All hypertensive patients included in this study were diagnosed with TTH by a neurologist in accordance with the criteria of the International Classification of Headache Disorders (ICHD-III beta) [29]. All participants were consulted by a cardiologist (hypertensive and non-hypertensive). The diagnosis of hypertension was established by a cardiologist in accordance with the criteria of the European Society of Cardiology and the European Society for Hypertension (2018) [30], the recommendations of the International Society for Hypertension Global Hypertension Practice Guidelines (2020) [31], and the Russian Society of Cardiology (2020) criteria [32].

This pilot study presents a part of the study “Clinical and Genetic Predictors of the OS (TTH and AH)”, registration No. 122030300108-6 dated 3 March 2022 [33]. The study was supported by the Grant of KrasMU to support the research of young scientists (No. 462-base, 12 July 2021). This pilot study was the part carried out from October 2020 to July 2022 [33]. 

The majority of the control individuals were anonymous blood donors; no information, except for sex, age and lifestyle (alcohol consumption, smoker status, salt intake, and level of physical activity) could be provided for these individuals. Informed consent to participate in this study was signed by all participants of this study (patients and healthy volunteers). Patients and healthy people did not receive any remuneration for participating in this study. Patients and healthy people (the control group) of this study did not pay for their participation in this study.

Altman’s nomogram method [34] in the online calculator MedStatistic [35] was used for the size of the sample calculation. We used significance level (alpha)—5% (0.05), and power (1-beta)—90% (0.9). The value of the expected frequency of OS (TTH in hypertensive patients) in the main group (OS group) was taken as 59.2% (taking into account our previous analysis [9]). The value of the expected frequency of the phenomenon in the control group (no headache in hypertensive patients) was taken as 15.0% (also taking into account our previous analysis [9]). The value of the standardized difference was 0.91. Therefore, according to the Altman nomogram used by us, the number of observed cases (participants in this pilot study) should have been at least 26 in each of the three comparable groups. The dropout rate was 10%. Thus, the sample size of each group had to be at least 29 participants each, and it was large enough to detect significant differences between comparable groups [33].

### 2.2. Study Population

Krasnoyarsk is one of the largest cities in Russia; it is the industrial, educational and cultural center of Eastern Siberia. Like all major cities in the world, the Krasnoyarsk city is subject to negative impacts on the environment. Its deterioration is facilitated by the fact that the city is the largest transport hub in Eastern Siberia; the presence of a number of large enterprises in the metallurgical, machine-building and chemical industries aggravates the situation. A significant share in the high level of atmospheric air pollution in Krasnoyarsk is automobile transport, the number of which is growing annually. The main substances that create high to very high levels of atmospheric pollution are benz(a)pyrene, formaldehyde, hydrochloride, suspended solids, and nitrogen dioxide [36]. Unfavorable environmental conditions increase the risk of developing cardiovascular diseases, including AH [37], as well as headaches, including TTH [38].

The city is located in Eastern Siberia, on both banks of the Yenisei River, at the junction of the West Siberian Plain, the Central Siberian Plateau and the Sayan Mountains, in a gorge formed by the northernmost spurs of the Eastern Sayan. The climate of Krasnoyarsk city is continental; softened by large water masses (the Krasnoyarsk reservoir), the Yenisei, which does not freeze in winter, and the surrounding mountains. Difficult climatic conditions also increase the risk of developing AH [37] and TTH [38].

The population of the city is 1,103,023 [39] (2022) people; it is the eighth most populous city in Russia. More than one and a half million people live in the Krasnoyarsk agglomeration, among which Caucasians predominate (more than 80%).

The total sample consisted of 91 participants who were residents of Krasnoyarsk, a large industrial city in Eastern Siberia. All participants in this study, including patients and healthy volunteers, were examined by a neurologist and therapist (or cardiologist) to confirm or exclude arterial hypertension and tension headache at the randomization stage. Based on the stratified randomization method, three observation groups were formed. The first group (or OS group) included 30 hypertensive patients (mean age—52.7 ± 5.7 years old) with TTH and AH. The second group (or AH group) included 30 hypertensive patients (mean age—53.6 ± 7.1 years old) without TTH. The third group (control) included 31 healthy participants (mean age—52.7 ± 6.7 years old). 

Criteria for inclusion in the first group (or OS group) [33]:

1. Residents of Krasnoyarsk city;
2. Caucasians;
3. Men and women;
4. Age: 40 to 65 years old [40];
5. Defined diseases: verified AH by a therapist or cardiologist and verified TTH by a neurologist;
6. Voluntary informed consent obtained from the participant of the study.

Criteria for inclusion in the second group (or AH group) [33]:

7. Residents of Krasnoyarsk city;
8. Caucasians;
9. Men and women;
10. Age: 40 to 65 years old [40];
11. Defined disease: verified AH by a therapist or cardiologist;
12. Voluntary informed consent obtained.

Criteria for inclusion in the third group (control group) [33]:

13. Residents of Krasnoyarsk city;
14. Caucasians;
15. Men and women;
16. Age: 40 to 65 years old [40];
17. Absence of AH, confirmed by a therapist or cardiologist;
18. Absence of TTH, confirmed by a neurologist;
19. Voluntary informed consent obtained.

Criteria for exclusion:

20. Migrants, small ethnic groups in Eastern Siberia;
21. Asians and Africans;
22. Children;
23. Young adults;
24. Other primary headaches (migraine, cluster headache, etc.) and secondary headaches;
25. Severe cognitive disorders or dementia;
26. Infectious diseases;
27. Diabetes mellitus;
28. Brain injury;
29. Stroke;
30. Epileptic seizures;
31. Renal and hepatic failure;
32. Chronic heart failure, class II and above, by the New York Heart Association (NIHA) Functional Classification;

### 2.3. DNA Isolation

Blood samples from participants were taken by venipuncture in the morning, on an empty stomach. Further, the blood samples were separated by centrifugation, after which the aliquots were stored in a refrigerator at −20 °C. Genomic DNA was isolated according to a standard protocol [41]. The primer was signed in accordance with the protocol of Applied Biosystems (USA). 

DNA was purified from whole blood with the Puregene^®^ Blood Core Kit C (QIAGEN Sciences, Hilden, Germany). DNA concentrations were measured with the NanoDrop^®^ ND-1000 Spectrophotometer (NanoDrop Technologies Inc., Wilmington, NC, USA) and the isolated material was used for the screening of selected genetic variants.

### 2.4. Genotyping by Quantitative Real-Time Polymerase Chain Reaction

TaqMan^®^ quantitative Real-Time Polymerase Chain Reaction (RT-PCR) (Applied Biosystems, Foster City, CA, USA) was used to determine the genotype of the SNPs rs2779249 and rs2297518 of the *NOS2* gene on chromosome 17q11 in case–control material using diagnostic equipment Rotor-Gene 6000 (Corbett Life Science, Australia) and technology allelic discrimination of TaqMan and fluorescent probes (Applied Biosystems, USA).

PCR plates with 2–5 ng DNA were prepared and dried overnight. TaqMan^®^ SNP Genotyping Assays (Applied Biosystems, USA) containing forward and reverse primers, as well as two 5′fluorescently labeled (VIC (2-chloro-7phenyl-1,4-dichloro-6-carboxy-fluorescein), FAM (carboxyfluorescein)) allele-specific probes were 1:1 diluted with 1× TE (Tris EDTA) buffer. For DNA elution, buffer was added to the tubes, resuspended, and incubated in a thermostat for 5 min at 65 °C, periodically vortexing. To remove the sorbent freed from DNA, the tubes were centrifuged for 1 min at a rotation speed of 12,000 rpm. The supernatant containing DNA was transferred into clean labeled tubes and stored at −20 °C [24].

### 2.5. Statistical Analysis

Statistical analysis related to the candidate gene approach included a simple comparison of the population frequencies of genotypes and SNPs in cases and controls using the Chi-square (χ^2^) criterion. This was in contrast to prospective genetic studies of allelic associations, in which phenotypic differences between genotypes of DNA sequence variants are compared using various parametric and nonparametric tests as needed [42]. 

Deviations from Hardy–Weinberg Equilibrium expectations were tested by the χ^2^ test. The gene counting method tested genotype distribution and allele frequencies. We used the χ^2^ test for the evaluation of differences in genotype distributions and allele frequencies between comparable and control groups. 

In this case–control study, the frequency of cases of OS and cases of AH were compared among individuals with normal alleles and individuals with allele variants, which allowed us to generate the odds ratio (OR). The most common type of allele variation of the SNP rs2779249 (chr17:26128581 C>A) was of a major allele C and a minor allele A. Thus, the genotype could be a homozygote for the major allele (CC), a heterozygote (CA) or a homozygote for the minor allele (AA). The most common type of allele variation of the SNP rs2297518 (chr17: chr17:27769571 G>A) was of a major allele G and a minor allele A. Thus, the genotype could be a homozygote for the major allele (GG), a heterozygote (GA) or a homozygote for the minor allele (AA).

Summarizing the data using a two-by-two contingency was used by us as the simplest method of estimating OR [43]. 

Three kinds of genotypes were transformed into two variables. The dominant model compared the CC genotype versus genotypes CA + AA for the SNP rs2779249 (chr17:26128581 C>A) and GG genotype versus genotypes GA + AA for the SNP rs2297518 (chr17: chr17:27769571 G>A). 

The recessive model compared CC + CA genotypes versus AA genotype for the SNP rs2779249 (chr17:26128581 C>A) and GG genotype versus genotypes GG + GA versus AA genotype for the SNP rs2297518 (chr17: chr17:27769571 G>A). 

An over-dominant model assumed the heterozygote had the strongest impact and compared genotypes CC + AA versus CA for the SNP rs2779249 (chr17:26128581 C>A) and GG + AA genotypes versus GA for the SNP rs2297518 (chr17: chr17:27769571 G>A). 

The multiplicative model hypothesized that CC, CA, and AA genotypes for the SNP rs2779249 (chr17:26128581 C>A) and GG, GA, and AA genotypes for the SNP rs2297518 (chr17: chr17:27769571 G>A) were associated with the lowest, the intermediate, and the highest risk of the OS, respectively, or they were associated with the highest, the intermediate, and the lowest risk, respectively [44]. 

We calculated the risk (OR, 95% confidential interval (CI)) for each model, including the risks of OS and AH [34,36]. *p*-values < 0.05 were considered as significant. 

## 3. Results

### 3.1. General Characteristic of Groups

The groups were comparable in terms of the age and sex of participants (*p*-value > 0.05). The mean age of AH onset in the study sample in the OS group and AH group were 40.6 ± 11.7 and 37.8 ± 11.5 years, respectively (*p*-value > 0.05); the mean age of TTH onset in the first group was 37.5 ± 10.4 years. The mean duration of AH anamnesis in the study sample in the first and second groups were 15.3 ± 11.2 years and 15.8 ± 9.9 years, respectively (*p*-value > 0.05).

Significant differences were not observed (*p*-value < 0.05) in traditional risk factors of TTH in hypertensive patients (OS group) and without TTH (AH group), and healthy participants (control group), including: alcohol consumption, smoker status, salt intake, and level of physical activity. 

Four main triggers for increasing blood pressure in hypertensive patients (OS and AH groups) were identified. However, there were no significant differences in three of the triggers (*p*-value > 0.05): psycho-emotional stress, intense physical activity, and sleep disorders. However, episodes of headache in hypertensive patients with TTH (OS group) were significantly more likely to provoke an increase in blood pressure compared to hypertensive patients without headache (*p*-value < 0.05).

### 3.2. Single-Nucleotide Polimorphism Analysis

In analyses of the frequencies of the SNP rs2779249 of the *NOS2* gene, compliance with the Hardy–Weinberg Equilibrium was found in the OS group (χ2 = 0.068; *p*-value = 0.97), AH group (χ2 = 0.3; *p*-value = 0.86) and the control group (χ2 = 0.894; *p*-value = 0.64). In addition, the frequencies of the SNP rs2297518 of this gene were compliant with the Hardy–Weinberg Equilibrium in the OS group (χ2 = 0.405, *p*-value = 0.82), AH group (χ2 = 0.37, *p*-value = 0.83), and the control group (χ2 = 0.08, *p* = 0.96).

Thus, two models were used to evaluate the obtained results: multiplicative (estimation of allele frequencies) and general (estimation of genotype frequencies). The allele frequencies and genotype distribution for rs2779249 and rs2297518 are demonstrated in Table 1 and Table 2, respectively.

The frequency of the minor allele A rs2779249 of the *NOS2* gene was significantly higher among patients with AH compared with healthy volunteers: 2.4 times in the OS group compared to the control group and 2.3 times in the AH group compared to the control group: 35.0% vs. 14.5% (*p*-value = 0.009) and 33.3% vs. 14.5% (*p*-value = 0.015), respectively. The frequency of the heterozygous genotype CA was significantly higher in the OS group compared to the control group (43.4% vs. 3.2%, *p*-value = 0.03). In addition, the frequency of the heterozygous genotype CA was statistically significantly higher in the AH group compared to the control group (40.0% vs. 3.2%, *p*-value = 0.045). The comparison of the frequencies of the recessive homozygous genotype AA was not possible, since no carriers of this genotype were identified among the healthy Siberian Caucasians (the control group).

The frequency of the minor allele A rs2297518 of the *NOS2* gene was significantly higher among patients with AH compared with healthy volunteers: 4.5 times in the OS group compared to the control group and 6.25 times in the AH group compared to the control group: 21.7% vs. 4.8% (*p*-value = 0.006) and 30.0% vs. 4.8% (*p*-value = 0.000233), respectively. The frequency of the heterozygous genotype GA was significantly higher in the OS group compared to the control group (30.0% vs. 9.7%, *p*-value = 0.046). In addition, the frequency of the heterozygous genotype GA was significantly higher in the AH group compared to the control group (46.7% vs. 9.7%, *p*-value = 0.001). The comparison of the frequencies of the recessive homozygous genotype AA was not possible, since no carriers of this genotype were identified among the healthy Siberian Caucasians (the control group).

The risks of OS and AH development depending on major (wild-type) alleles or minor (rare) and genotypes of rs2779249 and rs2297518 of the *NOS2* gene are presented in Table 3 and Table 4, respectively.

Therefore, the minor allele A of rs2779249 of the *NOS2* gene was associated with a risk of OS development (OR = 3.17 [95% CI: 1.31–7.67], *p*-value = 0.009) and AH (OR = 2.94 [95% CI: 1.21–7.15], *p*-value = 0.015) compared with the control group. The heterozygous genotype CA was associated with a risk of OS (OR = 1.87 [95% CI: 0.65–5.39], *p*-value = 0.03) and AH (OR = 1.63 [95% CI: 0.56–4.73], *p*-value = 0.045) compared with the control group. However, the association of this genotype with OS and AH was at the lower limit of statistical significance. Then, we compared the allele frequencies of the SNP rs2779249 of the *NOS2* gene between hypertensive patients with TTH and without headache (OS and AH groups, Table 3). However, no significant intergroup differences in the frequency of major allele C (65.0% vs. 66.7%) and minor allele A (35.0% vs. 33.3%) were found (*p*-value = 0.847) when assessing the frequencies of genotypes between the OS group and the AH group. There were also no significant differences (*p*-value = 0.962) between the OS group and the AH group for the heterozygous genotype CA (43.4% vs. 40.0%), the homozygous dominant genotype CC (43.4% vs. 46.7%) and homozygous recessive genotype AA (13.3% vs. 13.3%).

The minor allele A of rs2297518 of the *NOS2* gene was associated with a risk of OS development (OR = 5.44 [95% CI: 1.46–20.21], *p*-value = 0.006) and AH development (OR = 8.43 [95% CI: 2.33–30.46], *p*-value = 0.000223) compared with the control group. The heterozygous genotype GA was associated with OS risk (OR = 4.0 [95% CI: 0.96–16.61], *p*-value = 0.035) and AH (OR = 8.17 [95% CI: 2.03–32.79], *p*-value = 0.001) compared with the control group. However, the association of this genotype with OS and AH was at the lower limit of significance. In addition, we compared allele frequencies of the SNP rs2297518 of the *NOS2* gene between hypertensive patients with TTH and without headache (OS and AH groups, Table 4). However, no significant intergroup differences in the frequency of major allele G (78.3% vs. 70.0%) and minor allele A (21.7% vs. 30.0%) were found (*p*-value = 0.297) when assessing the frequencies of genotypes between the OS group and the AH group. There were also no significant differences (*p*-value = 0.398) between the OS group and the AH group for the heterozygous genotype GA (30.0% vs. 46.7%), the homozygous dominant genotype GG (63.3% vs. 46.7%), and homozygous recessive genotype AA (6.7% vs. 6.6%), respectively.

## 4. Discussion

Multiple variants of DNA sequences in the genome and multilevel regulation of gene expression and function indicate the complexity of the determinants of complex phenotypes (also known as OSs) [42,45] and such phenotypes are the result of additive effects and interactions between multiple SNPs with various genomic and environmental factors. There is a hypothesis that in OSs, the effect of the sizes of the alleles involved will vary and follow a gradient that varies from minimal or imperceptible to large and significant. At the same time, it is highly likely that only a few alleles will have a great influence on the development of this OS (TTH and AH) [4,6,42].

It is likely that many of the SNPs will have moderate effects, which in themselves may not be noticeable based on modern approaches to phenotyping and genetics. However, the newly introduced alleles, unlike wild-type alleles, are not characterized by evolutionary selective filtration and, consequently, minor alleles of these SNPs may have a larger clinical effect in OSs [42].

The gradient in the size of the clinical effect is partly related to the number of competing genetic and non-genetic (environmental) predictors that make a significant contribution to the development of the phenotype (for example, this OS—TTH and AH) [9]. As known, NO, produced by iNOS, performs important biological functions in the human body; it helps to transmit nerve signals, participates in suppressing inflammation, expands the lumen of blood vessels, has a bronchodilating effect, and so on. At the same time, dysfunctional induction of expression of this enzyme appears to be involved in the pathogenesis of many human diseases. In addition, it is important to remember that nitric oxide is a free radical and can damage cells. Therefore, iNOS expression is regulated quite strictly. It is known that modulation of iNOS expression at the transcriptional and post-transcriptional levels refers to the main mechanisms of regulation of the functional activity of this enzyme and its mediated synthesis of NO in response to environmental factors [46]. The treatment of overlap syndromes is adapted to active clinical manifestations and ranges from classical supportive therapy to the development of new therapeutic strategies. Such therapeutic strategies for TTH and AH may include prescribing medications that modulate the activity of iNOS [6,18]. The scientific and clinical interest in the presented results of our pilot study is also explained by the fact that DNA profiling of hypertensive patients and the identification of a group with a highly genetically determined risk of developing this OS can help to reduce the risk of irrational pharmacotherapy, drug abuse, and cerebrovascular complications [11,12,13].

Pilot associative genetic studies of this OS (TTH and AH) can be initiated by the desire to gain preliminary knowledge about the potential involvement of the SNP *NOS2* gene in the pathogenesis of this complex clinical phenotype, which is called the candidate gene approach [41,44]. An alternative approach is an unbiased study of a large number of genes and variants and, as a rule, the entire genome, as in genome-wide association studies, to identify associated alleles [47]. In the presented part of our large study of this OS, we used the first approach [33].

In addition, associative genetic studies of complex clinical phenotypes, which include this OS (TTH and AH), are usually developed based on two hypotheses of either “a common OS—a common variant”, or “a rare variant—a common OS” [48,49]. The first hypothesis suggests that OSs are the result of the cumulative effect of a sufficiently large number of major allelic variants of the SNPs of the candidate gene (genes), each of which has a moderate effect on the formation of a complex clinical phenotype. On the contrary, the second hypothesis suggests that minor allelic variants of the SNPs of the candidate gene (genes) with large effect sizes are the main determinants of the heritability of a complex clinical phenotype (or OS). Given that the expected sizes of the clinical effects of alleles in the genome may be continuous, it is expected that the combination of minor and common alleles will contribute to the heritability of OS. However, in this population, the major allele, despite its modest effects, may have a large proportion, which can be explained by the number of shifts, even if each minor allele may have a greater significance on the clinical effect [50].

As we know, the study of the mechanisms of development and the genetics of common Oss in general medical practice, cardiology, and neurology is receiving increasing attention. However, the genetic biomarkers of OS (TTH and AH) have not yet been studied enough. Associative gene studies of SNPs of the *NOS2* gene are rare and performed in regions with different climatic and geographical conditions of participants’ residence and with ethnic heterogeneity of the population, including Finland [17], Brazil [18], and China [19].

Taking into account all the above, the incentive for our chosen approach to conduct this pilot associative genetic study using the *NOS2* candidate gene is a priori knowledge of the potential participation of the isoform of iNOS encoded by this gene in the pathogenesis of the OS of interest common in the Caucasian population [6,9]. The choice of the design of this pilot study is explained by the fact that the candidate gene (genes) is analyzed using case–control studies or prospective allelic associations. Accordingly, the frequencies of genotypes to these SNPs (rs2779249 and rs2297518) of the *NOS2* genes were determined by us by genotyping, and not by direct sequencing, as an acceptable alternative variant of associative genetic research.

Thus, groups of scientists from Finland and Brazil have received conflicting results regarding the association of the rs2779249 gene *NOS2* and AH. Therefore, Nikkari et al. [17] found that the carriage of the A allele (CA + AA genotypes) of rs2779249 of the *NOS2* gene induces the risk of developing AH compared to the carriage of the CC genotype (OR = 1.47, 95% CI = 1.08–2.01, *p*-value = 0.015) in the Finnish population, while Oliveira-Paula et al. [18] did not find such an association. According to the results, the carriage of the CA и AA genotypes rs2779249 of the *NOS2* gene did not affect the risk of developing AH (OR = 0.88, 95% CI = 0.43–1.76, *p*-value = 0.718 and OR = 0.93, 95% CI = 0.37–2.39, *p*-value = 0.883, respectively) in the Brazilian population.

Later, Zhai et al. [19] showed that the carriage of the A allele (rs2779249) of the *NOS2* gene induces the risk of developing AH in the additive model (OR = 1.27, 95% CI = 1.12–1.44), in the dominant model (OR = 1.31, 95% CI = 1.09–1.59), and in the recessive model (OR = 1.68, 95% CI = 1.28–2.19) in the Chinese population. According to the results of this study, the frequency of the minor allele A (rs2297518) of this gene was significantly higher among patients with hypertension compared to the control group (13.0% vs. 8.7%, *p*-value < 0.0001), and the frequency of carriage of the AA genotypes (7.9% vs. 4.7%) and GA (10.1% vs. 7.9%) was also significantly higher among patients with hypertension compared with the control (*p*-value = 0.0006). In addition, the authors [19] built genetic models, obtaining a significant association of rs2297518 with AH in additive (OR = 1.35 [95% CI: 1.15–1.57], *p*-value < 0.01) and dominant (GG vs. GA + AA: OR = 1.52 [95% CI: 1.21–1.91], *p*-value < 0.01) models in the Chinese population (Asians). Our study demonstrated comparable results in the European population (Caucasians living in Eastern Siberia); we managed to construct an additive genetic model (OR = 8.17 [95% CI: 2.03–32.79], *p*-value = 0.001).

**Figure 2:**
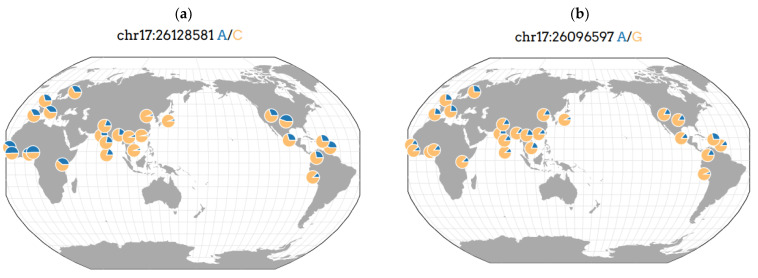
Geography of genetic variant rs2779249 (**a**) and rs2297518 (**b**) of the *NOS2* gene in the world [51]. Note: blue color—minor allele; orange color—major allele.

Our pilot study is the first to research the association of two SNPs (rs2779249 and rs2297518) of the *NOS2* gene (Figure 2a,b [51]) with OS (TTH and AH) in Caucasians of a large industrial city from Eastern Siberia (Krasnoyarsk, Russia). In addition, this is the first publication describing these SNPs of the *NOS2* gene as a prognostic biomarker (predictor) of OS in adults [4]. It demonstrated that the minor A allele of SNP rs2779249 was statistically significantly associated with a risk development of AH (OR = 2.94, 95% CI = 1.21–7.15, *p*-value = 0.015) and OS (OR = 3.17, 95% CI = 1.31–7.67, *p*-value = 0.009) in Caucasians of Eastern Siberia (Russia). In addition, the minor A allele of SNP rs2297518 was statistically significantly associated with a risk development of AH (OR = 8.43, 95% CI = 2.33–30.46, *p*-value = 0.000223) and OS (OR = 5.44, 95% CI = 1.46–20.21, *p*-value = 0.006) in our study. At the same time, there was a trend towards a higher risk of developing this overlap syndrome compared with AH, although these differences did not reach statistical significance.

The data from this pilot study significantly complement the earlier study of the role of SNP rs3782218 of the *NOS1* gene in Caucasians of Eastern Siberia [33], but do not agree with the results of the Oliveira-Paula et al. (2013) [18] study performed in Brazil (South America). However, our results are comparable to those of Nikkari et al. (2015) [17] in the Finnish population (Northern Europe) and Zhai et al. (2018) [19] in the Chinese population (Asia). Interestingly, the risk of OS (TTH and AH) was higher in Russia (OR = 3.17, 95% CI = 1.31–7.67) compared to Finland (OR = 1.47, 95% CI = 1.08–2.01) [17] and China (OR = 1.31, 95% CI = 1.09–1.59) [19].

We understand that an increase in the sample size of this pilot study and the inclusion of Krasnoyarsk city residents who are representatives of other ethnic and racial groups (for example, Asians living in Eastern Siberia) could complement the existing picture of the distribution of alleles and genotypes. Of course, the results of this pilot study do not allow us to draw general conclusions about the strength of the association of two SNPs (rs2779249 and rs2297518) of the *NOS2* gene with a genetic predisposition to this OS in Eastern Siberia as a whole. However, the data obtained on dozens of samples explain the importance and expediency of continuing research on these SNPs in the future. At the same time, it is necessary to take into account the difference in the distribution of the studied genetic biomarkers of this OS in different populations, including Caucasian, Asian, and African.

As is widely known, the frequency of allelic variants of SNPs rs2779249 and rs2297518 of the *NOS2* gene are variable in populations of the European, Asian and African regions (Figure 2a,b [51]). In addition, they may differ in different ethnic groups. Previously conducted associative genetic studies demonstrate that the frequency of allelic variants of genes responsible for a predisposition to MFDs can be highly variable both in mixed (ethnically and/or racially heterogeneous) populations of Western and Eastern Europe, and in a mixed population of Russia. According to the available data, the gene pool of the population of Eastern Siberia is less susceptible to the “drift of genes” from the countries of Western and Eastern Europe and, conversely, is more susceptible to the “drift of genes” from Asian countries. This was demonstrated in the previously published results of associative genetic studies of other MFDs in the population of the large Siberian Federal District of Russia [52].

This is important to remember, because a simple extrapolation of the results of rs2779249 and rs2297518 associative genetic studies of the *NOS2* gene conducted in remote regions of the world to the population of Eastern Siberia can lead to significant diagnostic errors and false conclusions. This point of view is most relevant for the *NOS2* gene, which encodes an inducible isoform of the enzyme of the NOS system, the level of expression of which depends on environmental factors (geographical, climatic, nutritional, industrial, etc.). Therefore, the results of our pilot study complement the overall still unclear picture of the role of the studied SNPs of the *NOS2* gene on the risk of OS. It is possible that an increase in the sample size in further associative genetic studies may provide new information about the frequency of rare homozygous (AA) genotypes of these SNPs in Caucasians living in a large industrial city in Eastern Siberia with an unfavorable ecology.

In addition, the expression of iNOS is influenced by external environmental factors, including climatic, geographical, and industrial ones. Krasnoyarsk is a large industrial city in Eastern Siberia and is one of the most unfavorable cities in the world according to the results of monitoring its environment and the content of industrial exotoxins in its air and soil [53]. We do not rule out the influence of these factors in inducing iNOS overexpression in the studied population. However, in comparable studies carried out in Finland and China, we did not find any information about the environmental situation in the studied regions.

## 5. Limitations

A limitation of this study is the impossibility of constructing a recessive model (AA/CC + CA for the SNP rs2779249, and AA/GG + GA for the SNP rs2297518), since the recessive homozygous genotypes (AA) were not found among healthy Siberian Caucasians (the control group).

Another limitation may be the absence of patients with TTH without AH as a comparable group. This is due to the fact that the purpose of our study was to search for a new biomarker of OS (TTH and AH) [6].

Carriers of the AA haplotype rs2779249 of the *NOS2* gene showed a higher risk of developing other primary headaches: migraine (OR = 2.65 [95% CI: 1.34–5.22], *p*-value = 0.0027) [10,54,55] in the Brazilian population, and cluster headache (χ2 = 5.21; *p*-value = 0.022) [56] in the Swedish population. However, we did not find studies of these SNPs in patients with TTH, which is of undoubted scientific clinical interest.

In addition, an approach using candidate gene(s) in a population of a prospective study design may yield more reliable results than the case–control study design we used. In addition, the sample size and characteristics of the studied groups of patients (OS, AH without TTH, and TTH without AH) may be key components of the design of a new (prospective) study of this complex clinical phenotype.

## 6. Conclusions

Our pilot study demonstrated that the SNPs rs2779249 (chr17:26128581 C>A) and rs rs2297518 (chr17: chr17:27769571 G>A) of the *NOS2* gene could be biomarkers (predictors) for the risk of OS (AH and TTH) development in Caucasian patients of a large industrial city from Eastern Siberia.

These data indicate that the studied SNPs in the *NOS2* gene can alter the level of iNOS expression and NO synthesis in the endothelium of peripheral and cerebral vessels. It is likely that this is influenced by adverse environmental factors (climatic, geographical, and industrial), and that these disorders may be reflected in the form of changes in clinical signs in hypertensive patients (for example, in the development of this OS). However, in the future, it is necessary to conduct research on the interaction between genetic and environmental factors as part of new research.

## Figures and Tables

**Table 1:** Allele frequencies and genotype distribution of rs2779249 of the *NOS2* gene.

Allele, Genotype	Overlap Syndrome Group	Arterial Hypertension Group	Control Group	*p*-Value
C	39 (65.0%)	40 (66.7%)	61 (98.4%)	0.009 *, 0.015 **
A	21 (35.0%)	20 (33.3%)	1 (1.6%)	
CC	13 (43.35%)	14 (46.7%)	30 (96.8%)	0.03 *, 0.045 **
CA	13 (43.35%)	12 (40.0%)	1 (3.2%)	
AA	4 (13.3%)	4 (13.3%)	0 (0%)	

Note: * *p*_1_-value—overlap syndrome group vs. control group; ** *p*_2_-value—arterial group vs. control group.

**Table 2:** Allele frequencies and genotype distribution of rs2297518 of the *NOS2* gene.

Allele, Genotype	Overlap Syndrome Group	Arterial Hypertension Group	Control Group	*p*-Value
G	47 (78.3%)	42 (70.0%)	59 (95.2%)	0.006 *, 0.000223 **
A	13 (21.7%)	18 (30.0%)	3 (4.8%)	
GG	19 (63.3%)	14 (46.7%)	28 (90.3%)	0.035 *, 0.001 **
GA	9 (30.0%)	14 (46.7%)	3 (9.7%)	
AA	2 (6.7%)	2 (6.6%)	0 (0%)	

Note: * *p*_1_-value—overlap syndrome group vs. the control group; ** *p*_2_-value—arterial group vs. the control group.

**Table 3:** Odds ratio (OR) of tension-type headache and arterial hypertension overlap syndrome depending on alleles and genotypes of rs2779249 of the *NOS2* gene.

Allele, Genotype	χ^2^	*p*-Value	OR	95% Confidential Interval
Overlap Syndrome Group vs. Control Group				
C	6.9	0.009	0.32	0.13–0.76
A			3.17	1.31–7.67
CC	7.027	0.03	0.31	0.11–0.9
CA			1.87	0.65–5.39
AA			-	-
Arterial Hypertension Group vs. Control Group				
C	5.958	0.015	0.34	0.14–0.83
A			2.94	1.21–7.15
CC	6.192	0.045	0.36	0.12–1.03
CA			1.63	0.56–4.73
AA			-	-
Overlap Syndrome Group vs. Arterial Hypertension Group				
C	0.037	0.847	0.93	0.44–1.98
A			1.08	0.51–2.29
CC	0.077	0.962	0.87	0.32–2.42
CA			1.15	0.41–3.2
AA			1.0	0.23–4.43

**Table 4:** Odds ratio (OR) of tension-type headache and arterial hypertension overlap syndrome depending on alleles and genotypes of rs2297518 of the *NOS2* gene.

Allele, Genotype	χ^2^	*p*-Value	OR	95% Confidential Interval
Overlap Syndrome Group vs. Control Group				
G	7.578	0.006	0.18	0.05–0.67
A			5.44	1.46–20.21
GG	6.709	0.035	0.19	0.05–0.75
GA			4.0	0.96–16.61
AA			-	-
Arterial Hypertension Group vs. Control Group				
G	13.547	0.000223	0.12	0.03–0.43
A			8.43	2.33–30.46
GG	13.772	0.001	0.09	0.02–0.38
GA			8.17	2.03–32.79
AA			-	-
Overlap Syndrome Group vs. Arterial Hypertension Group				
G	1.087	0.297	1.55	0.68–3.54
A			0.65	0.28–1.47
GG	1.845	0.398	1.97	0.7–5.54
GA			0.49	0.17–1.41
AA			1.0	0.13–7.6

Note: TTH—tension-type headache; AH—arterial hypertension.

## Data Availability

Not applicable.
